# Mechanisms of Resistance to Decitabine in the Myelodysplastic Syndrome

**DOI:** 10.1371/journal.pone.0023372

**Published:** 2011-08-17

**Authors:** Taichun Qin, Ryan Castoro, Samih El Ahdab, Jaroslav Jelinek, Xiaodan Wang, Jiali Si, Jingmin Shu, Rong He, Nianxiang Zhang, Woonbok Chung, Hagop M. Kantarjian, Jean-Pierre J. Issa

**Affiliations:** 1 Department of Leukemia, The University of Texas MD Anderson Cancer Center, Houston, Texas, United States of America; 2 Harbin Institute of Hematology & Oncology, Harbin, China; University of Nebraska Medical Center, United States of America

## Abstract

**Purpose:**

The DNA methylation inhibitor 5-aza-2′-deoxycytidine (DAC) is approved for the treatment of myelodysplastic syndromes (MDS), but resistance to DAC develops during treatment and mechanisms of resistance remain unknown. Therefore, we investigated mechanisms of primary and secondary resistance to DAC in MDS.

**Patients and Methods:**

We performed Quantitative Real-Time PCR to examine expression of genes related to DAC metabolism prior to therapy in 32 responders and non-responders with MDS as well as 14 patients who achieved a complete remission and subsequently relapsed while on therapy (secondary resistance). We then performed quantitative methylation analyses by bisulfite pyrosequencing of 10 genes as well as Methylated CpG Island Amplification Microarray (MCAM) analysis of global methylation in secondary resistance.

**Results:**

Most genes showed no differences by response, but the CDA/DCK ratio was 3 fold higher in non-responders than responders (P<.05), suggesting that this could be a mechanism of primary resistance. There were no significant differences at relapse in DAC metabolism genes, and no DCK mutations were detected. Global methylation measured by the LINE1 assay was lower at relapse than at diagnosis (P<.05). On average, the methylation of 10 genes was lower at relapse (16.1%) compared to diagnosis (18.1%) (P<.05).MCAM analysis showed decreased methylation of an average of 4.5% (range 0.6%–9.7%) of the genes at relapse. By contrast, new cytogenetic changes were found in 20% of patients.

**Conclusion:**

Pharmacological mechanisms are involved in primary resistance to DAC, whereas hypomethylation does not prevent a relapse for patients with DAC treatment.

## Introduction

The myelodysplastic syndrome (MDS) encompasses a diverse group of clonal hematopoietic disorders united by ineffective production of blood cells and varying risks of transformation to acute myelogenous leukemia (AML). MDS is typically a disease of older adults [1]. Epigenetic deregulation plays an important role in the pathogenesis of MDS. Hypermethylation of CpG islands in the promoter of tumor-associated genes and their consequent silencing are important in the pathogenesis of MDS [2],[3]. Reversal of aberrant methylation leads to re-expression of silenced tumor suppressor genes and appears to be important in the response and prognosis of patients treated with DAC [4]. The prototypical DNA methyltransferase inhibitor 5-aza-2′-deoxycytidine (decitabine, 5-aza-dC, DAC) and 5-azacytidine (AZA) have been approved by the Food and Drug Administration (FDA) as antitumor agents for the treatment of MDS. Low-dose decitabine has been studied recently in multiple clinical trials and has been shown to be effective for treatment of MDS [4], [5].

In clinical trials, it was found that a number of patients do not respond to DAC initially (primary resistance) and most patients who initially respond to DAC treatment, eventually relapse (secondary resistance) despite continued DAC therapy [4]. Mechanisms of primary and secondary resistance to existing DNA methylation inhibitors have not been determined. Most primary mechanisms of resistance to cytosine analogues (NAs) are based on metabolic pathways [6], [7]. A primary mechanism is an insufficient intracellular concentration of NA triphosphates, which may result from multiple factors including insufficient uptake through membrane transporters, deoxycytidine kinase (dCK) deficiency, increased deamination by cytidine deaminase (CDA), or high dNTP pools.

We previously found that mechanisms of naturally occurring resistance to DAC in vitro in a panel of cancer cell lines was primarily due to insufficient intracellular triphosphate, resulting from DCK mutations or aberrant gene expression [8]. Here we tested mechanisms of primary and secondary resistance to DAC in vivo in MDS patients.

## Materials and Methods

### Patients

Adults with a diagnosis of MDS who were referred to MD Anderson Cancer Center were enrolled in the study after informed consent was obtained according to institutional guidelines and in accordance with the Declaration of Helsinki. Patients were categorized for MDS risk at the initiation of decitabine therapy and at the time of failure of decitabine according to the IPSS [9] and to the MDACC risk model [10].Bone marrow and/or peripheral blood cells were collected from consenting patients according to institutional guidelines and an IRB approved protocol. Genomic DNA was isolated using DNA STAT-60 reagent (Iso Tex Diagnostics, Friendswood, TX) according to the manufacturer's instructions.

### Bisulfite-pyrosequencing for methylation analysis

Bisulfite treatment was performed as reported previously [11], [12]. Bisulfite-treated DNA (40–80 ng) was amplified with gene-specific primers in a 2-step polymerase chain reaction (PCR). Primer sequence for 5 genes and LINE1 analyzed are shown in Table S1. The second step of PCR was used to label single DNA strands with biotin using a universal primer tag [13] or gene-specific primers biotinylated at the 5′end. We measured levels of DNA methylation as the percentage of bisulfite-resistant cytosines at CpG sites by pyrosequencing with the PSQ HS 96 Pyrosequencing System (Biotage, Charlottesville, VA) and Pyro Gold CDT Reagents (Biotage) as previously described [13].

### Quantitative reverse transcription-PCR

Real-time quantitative reverse transcription-PCR was done with the ABI 7700 Sequence Detector (Applied Biosystems).We used commercially available primers sets with minor groove binder probe for genes and GAPDH as an internal control (Applied Biosystems). Reactions for quantitative reverse transcription-PCR were done with the TaqMan universal PCR Master Mix kit (Applied Biosystems) in 96-well plates. Each sample was measured in triplicate. PCR was run using the following conditions: an initial denaturation step of 95°C for 10 min followed by 40 cycles at 95°C for 15 s and 60°C for 1 min. Data were analyzed with ABI Prism 7000 SDS software (Applied Biosystems).

### Sequencing of DCK

We performed RT-PCR on the full coding region of DCK and amplified a PCR product to directly sequence DCK gene mutations. The forward primer 5′ TCTTTGCCGGACGAGCTCTG ′ and reverse primer 5′ CAGGCAGCCAAATGGTTC 3′, cover the full coding region from exon 1 to exon 7. The length of this PCR product is 858 bp. PCR was run using the following conditions: an initial denaturation step of 95°C for 5 min followed by 40 cycles at 95°C for 15 s, 60°C for 30 s, and 72°C for 60 s.

### Methylated CpG island microarray (MCAM)

We used DNA from the bone marrow samples of 4 patients with MDS obtained at the time of initial diagnosis and at the time of first relapse. Methylated CpG island amplification was performed as described [14]. Amplicons from patients with MDS after relapse were labeled with the Cy5 dye and cohybridized against amplicons from patients at diagnosis labeled with the Cy3 dye on Agilent Technologies 4×44 K custom DNA microarrays (Agilent, Santa Clara, CA) as described previously [15]. Dye swaps were preformed for comparison. This method allows parallel analysis of 42222 probes corresponding to 9008 autosomal genes. The probes on the array were selected to recognize *SmaI*/*XmaI* fragments, mostly around gene transcription start sites. We used normalized signal intensity based on Agilent software to perform microarray hybridization analysis as previously described [15]. We used probes located outside of *SmaI*/XmaI fragments (length up to 10 kb) for normalization and background calculation. The signal intensity for the probes within the *SmaI*/XmaI fragments was adjusted for background and analyzed for the ratio between Cy3 and Cy5 signals. The ratios of hybridization intensities were adjusted by using Lowess normalization.

### Statistical Analysis

Statistical differences between different patient groups were analyzed by Mann-Whitney nonparametric t-test. Statistical differences between same patient groups were analyzed by paired t test. 2-sided P values<0.05 were considered statistically significant. All calculations were done using GraphPad Prism 4.0 (GraphPad Software Inc.). The stata 10 was used for univariate and multivariate analysis of the correlation of biological features with drug response.

## Results

### Patients studied

We examined patients with primary resistance (never responded) and secondary resistance (responded then relapsed) to DAC. For primary resistance, we included 32 patients who were randomized to receive DAC 20 mg/m^2^ intravenously over 1 hour daily for 5 days. For the secondary resistance study, we included 14 patients from a different clinical trial who were randomized to receive DAC in 20 mg/m^2^ intravenously over 1 hour daily for 5 days. The patients were considered to have not responded only after having received at least 3 courses of therapy. Patient characteristics are described in Table 1. There were no statistically significant difference in disease characteristics between responders and non-responders in patients with primary resistance, but bone marrow blast (%) in patients with secondary resistance at the diagnosis was lower than at relapse (7% vs 16%, P<0.05).

**Table 1:** Patients studied for secondary resistance.

Clinical Characteristic	Primary Resistance, Median (Range)		Secondary Resistance, Median (Range)	
	Responders (N = 16)	Non-responders (N = 16)	Diagnosis (N = 30)	Relapse (N = 30)
Age, years	70 (56–83)	68 (50–84)	69 (56–81)	
Males, n(%)	14 (87)	9 (56)	20 (67)	
Overall survival duration, months			18 (8–36)	
Time from diagnosis to relapse, months			11 (3–24)	
Time from relapse to death, months				4 (2–26)
Bone Marrow Blasts (%)	4 (0–15)	12 (1–70)	7 (0–15)	16 (2–55)*
White Blood Cells, 10^3^/µL	13.1 (3.5–61.5)	8.6 (0.1–44.4)	3.8 (0.9–83.5)	2.8 (0.7–145.4)
Hemoglobin, g/dL	11.2 (8.1–14.7)	9.5 (8.3–11.5)	9.7 (6.7–14.1)	9.7 (7.4–13.6)
Platelets, 10^3^/µL	100 (8–262)	43 (4–129)	75 (10–392)	43 (5–486)
**Karyotype, n (%)**				
Good	9 (56)	4 (24)	15 (30)	12 (25)
Intermediate	2 (12)	3 (18)	11 (36)	12 (25)
Poor	2 (12)	6 (36)	4 (13)	6 (20)
Unclassified	3 (18)	3 (18)	0	0
**IPSS risk category, n (%)**				
Low	3 (19)	2 (13)	6 (20)	
Intermediate-1	4 (25)	2 (13)	9 (30)	
Intermediate-2	4 (25)	4 (25)	8 (27)	
High	1 (6)	4 (25)	4 (14)	
Unclassified	4 (25)	4 (25)	5 (17)	

*P<0.05.

### DAC metabolism gene expression in primary resistance

**Figure 1:**
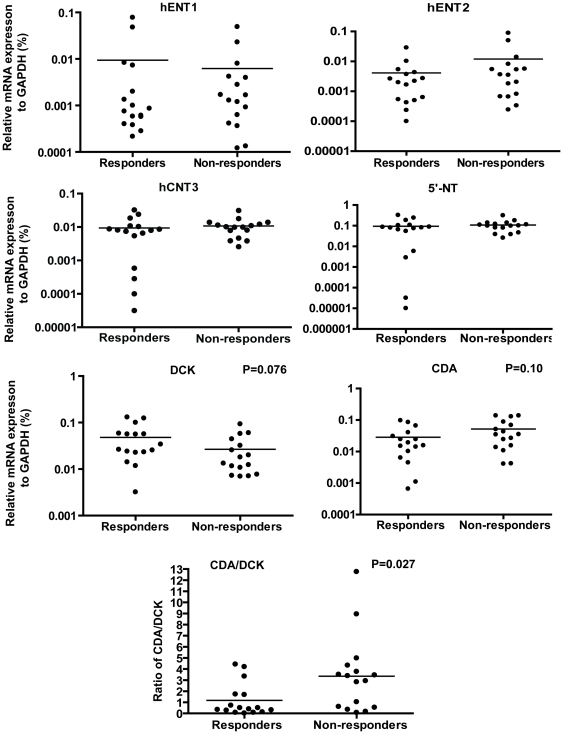
DAC metabolism gene expression in primary resistance. mRNA expression of genes related to DAC metabolic pathways including hENT1, hENT2, hCNT3, 5′-NT, CDA and DCK were measured by Quantitative Real-Time PCR in 16 responders and 16 non-responders with primary resistance to DAC. GAPDH was used as an internal control. Statistical analysis of the differences in gene expression was performed by unpaired t tests.

We compared the expression of a group of genes related to the metabolism of DAC including hENT1, hENT2, hCNT3, DCK, CDA and 5 ′-NT between responders and non-responders. Individually, none of the genes were significantly different between responders and non-responders (Figure 1). There was a trend for DCK expression to be lower in non-responders (P = 0.076, Figure 1). There was also a trend for CDA, which inactivates DAC, to be higher in non-responders (P = 0.10) (Figure 1). We therefore examined the ratio of CDA/DCK and found that it was 1.2±0.37 in responders, but significantly increased to 3.4±0.85 in non-responders with the primary resistance (P = 0.027) (Figure 1). These results suggest that primary resistance to DAC may be due to increased deamination and decreased phosphorylation in a subset of patients with primary resistance.

### DAC metabolism gene expression in secondary resistance

**Figure 2:**
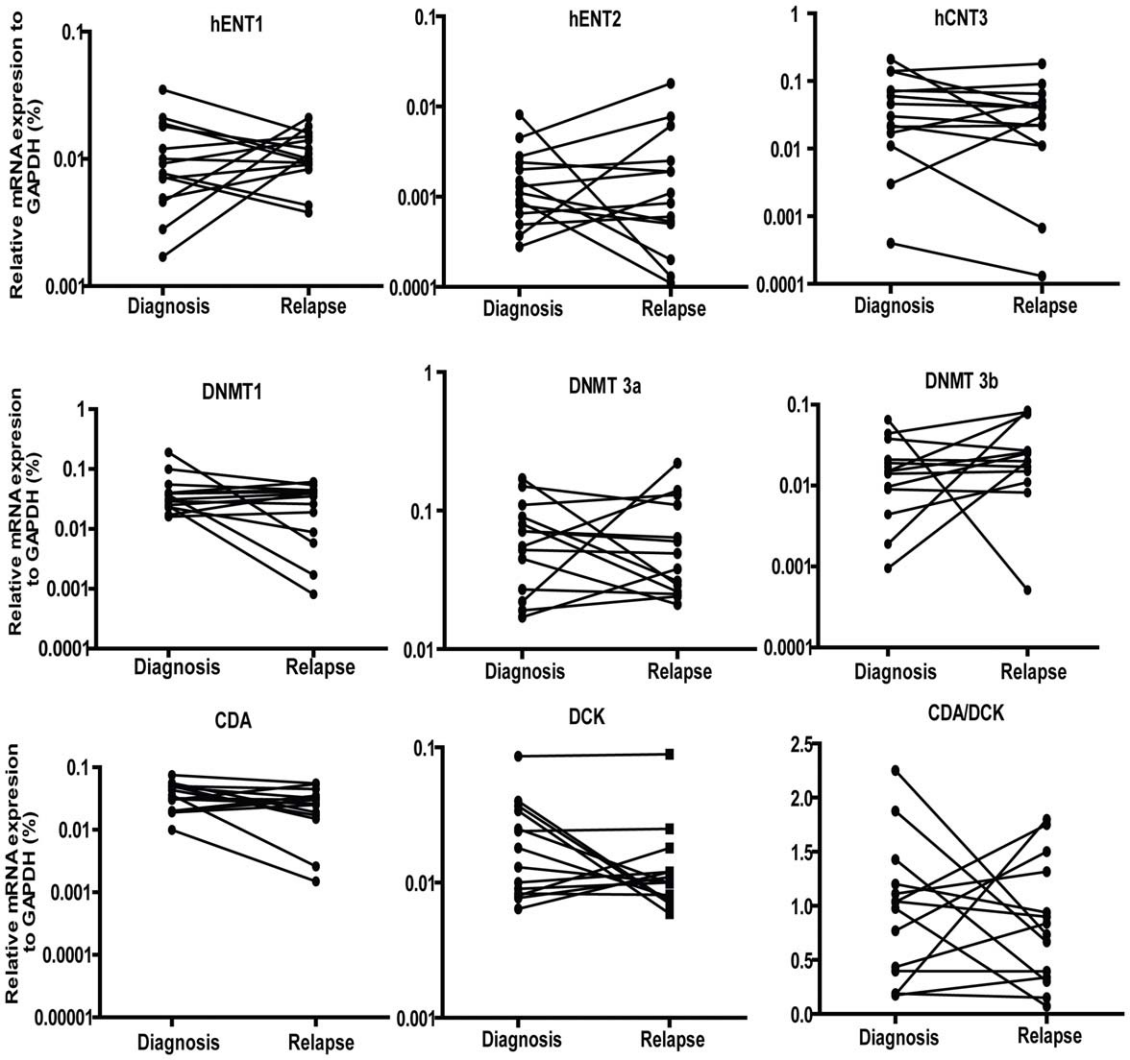
DAC metabolism gene expression in secondary resistance. mRNA expression of hENT1, hENT2, hCNT3, DCK, CDA, MDR1, DNMT1, DNMT3a, and DNMT3b were measured by quantitative real-time PCR between 14 MDS patients at diagnosis and after relapse. GAPDH was used as an internal control. Statistical analysis of the difference of gene expression was performed by paired t test.

Using quantitative real-time PCR, the mRNA expression levels of genes related to DAC metabolism including hENT1, hENT2, hCNT3, DCK, CDA, MDR1 were measured at diagnosis and at relapse. Detectable amounts of all nine genes were found in all 14 samples. There was no significant difference in mRNA expression of these genes between diagnosis and relapse. There was also no significant difference in the CDA/DCK ratio (Figure 2). We next measured mRNA expression of DNA methyltransferase genes DNMT1, 3a and 3b and found that there was no significant difference in gene expression between diagnosis and relapse (Figure 2). Furthermore, we sequenced the DCK coding region for mutations in patients after relapse. We obtained 16 patient samples after relapse, extracted RNA, and synthesized cDNA. We used the primers that covered the full coding region of DCK. No mutations were detected in the coding region of all the samples. Thus, DCK mutations or mRNA expression of DAC metabolism genes do not explain secondary resistance.

### DNA methylation analysis at relapse

**Figure 3:**
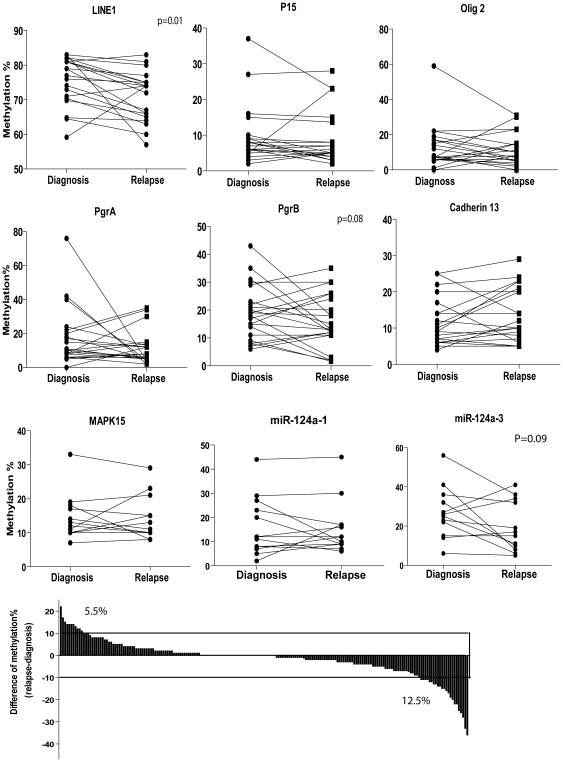
Measurement of gene methylation in MDS patients with secondary resistance. A. Pyrosequencing of gene methylation. We measured methylation of LINE1 and five genes in 20 MDS patients between diagnosis and relapse. Paired t test was used to measure the difference in methylation levels. B. Difference of methylation between diagnosis and relapse. We calculated the difference in methylation between diagnosis and relapse and highlight changes above a 10% cut-off.

We next asked whether patients who relapsed after an initial response to DAC showed any significant difference in gene methylation. We studied global methylation of LINE1 and the following genes: CDKN2B (p15^INK4b^), PGRA, PGRB, OLIG2, NOR1, CDH13, MAPK15, miR-124a-1, and miR-124a-3 by bisulfite pyrosequencing in 12–20 MDS patient samples. Methylation of those genes has been described in leukemia. For example, P15 is inactivated selectively in leukemias and gliomas and seems to constitute an important tumor suppressor gene loss in these neoplasms [16]; CDH13 expression by aberrant promoter methylation occurs at an early stage in CML pathogenesis [17]; Extensive methylation of PGRA and PGRB was also observed in leukemia samples [18]; *miR-124-1* is a tumor suppressor microRNA (miR). Epigenetic deregulation of miR is implicated in haematological malignancies [19]. Paired t-test analysis comparing methylation levels at baseline and relapse showed that there was hypomethylation of LINE1 (P = 0.01) at relapse, a trend for hypomethylation of PGRB (P = 0.08) and miR-124a-3 (P = 0.08) at relapse, and no significant differences in other genes (Figure 3A). On average, methylation density was significantly decreased from 18.1%±20.5% at diagnosis to 16.1%±18.4% at relapse by Wilcoxon signed rank test (P = 0.02). All changes in DNA methylation status in individual patients between diagnosis and relapse are shown in Figure 3B. Considering a 10% difference as significant, 11/199 (5.5%) measurements showed increased methylation after relapse, 25/199 (12.5%) showed decreased methylation, and 164 showed no differences (Figure 3B). Thus, analysis of these genes suggested that patients had more hypomethylation after relapse.

**Figure 4:**
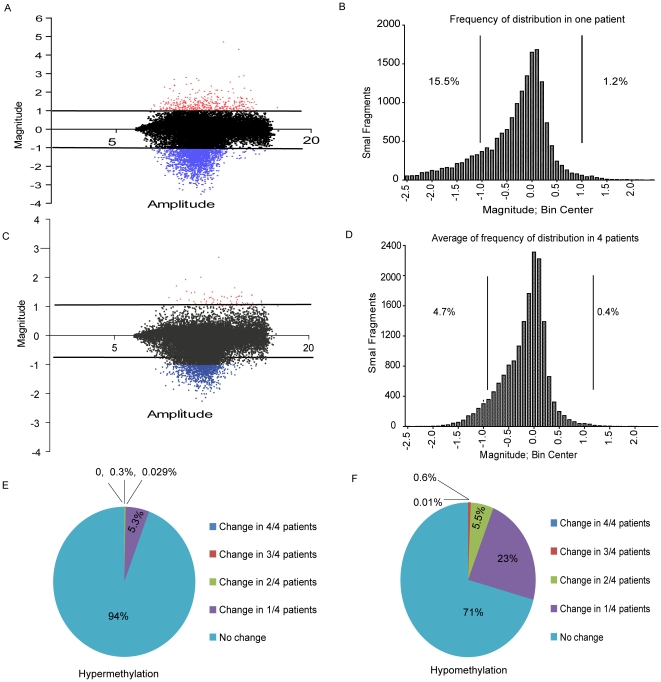
MCAM analysis of secondary resistance. A. Representative plot of A–M MCAM analysis in one patient. The plot shows Amplitude (A) = ½ (log_2_ R×G), and Magnitude (M) = log_2_(R/G). The red, black, and blue spots indicate probes hypermethylated, unchanged, and hypomethylated in MDS patients, respectively. B. Frequency of distribution of *SmaI* fragments in one patient. Values below −1 indicate hypomethylation at relapse, while values above 1 indicate hypermethylation. C. Representative plot of A–M MCAM analysis in 4 patients. We averaged A and M in four patients and performed A–M plot. D. Frequency of distribution of *SmaI* fragments in 4 patients. We averaged the value of *SmaI* fragments in four patients and performed analysis of frequency of distribution. E. Pie diagram of hypermethylated genes at relapse. We calculated the percentage of genes that are not hypermethylated, hypermethylated in 1 patient, or commonly hypermethylated in 2, 3, or 4 patients at relapse, respectively. Pie diagram was performed based on the percentage of these genes. F. Pie diagram of hypomethylated genes at relapse. We calculated the percentage of genes that are not hypomethylated, hypomethylated in 1 patient, or commonly hypomethylated in 2, 3, or 4 patients at relapse, respectively. Pie diagram was performed based on the percentage of these genes.

Next, we analyzed genome wide methylation by MCAM [14] in 4 patients at diagnosis and relapse. In each case, we cohybridized DNA from diagnosis and relapse in the same slide. A representative M-A plot of the data in one patient is shown in Figure 4A. We calculated the frequency of methylation change in *Sma*I fragments in that patient, and found that 15.5% of *SmaI* fragments were hypomethylated while 1.2% were hypermethylated (Figure 4B). A M-A plot averaging of the data in all 4 patients is shown in figure 4C. On average, 4.7% of *SmaI* fragments were hypomethylated compared with 0.4% , which were hypermethylated (Figure 4D).

We next analyzed the data for *SmaI* sites within 1 kb of transcription starting sites, and used a more stringent criterion for hyper/hypomethylation (M value>1.5 or <−1.5) to reduce false positives. In this analysis of 6832 genes, hypomethylation at relapse averaged 4.5% (range 0.6%–9.7%), while hypermethylation averaged 0.9% (range 0.1%–2.0%). Among these genes, hypermethylation in 2 or more cases was rare (0.33%) and hypomethylation was seen in 6% of loci in 2 or more cases. (Figure 4E, 4F). A list of genes modified in 2 or more patients is shown in Table S2, S3, S4.

**Figure 5:**
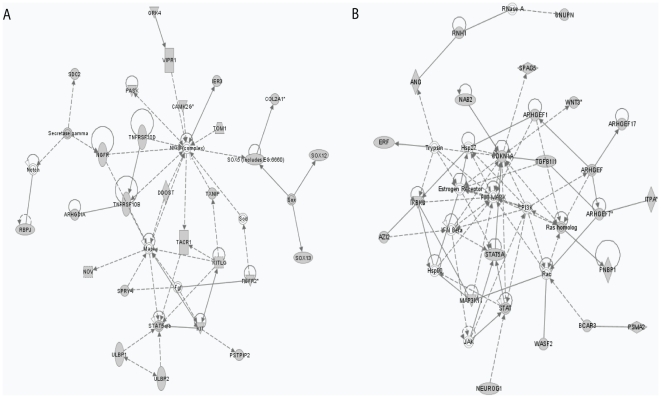
The most prominently affected gene networks generated by Ingenuity Pathway Analysis. A Genes hypomethylated in 2 or more patients in this network are responsible for lipid metabolism, small molecule biochemistry, and cancer. B Genes hypomethylated in 2 or more patients in this network are responsible for cell death, cancer, DNA replication, recombination and repair.

Ingenuity analysis of those genes commonly hypomethylated in 2 or more patients showed enrichment of network functions in lipid metabolism, small molecule biochemistry, cell cycle, genetic disorders, cancer, DNA replication, recombination, and repair. Ingenuity analysis of those genes commonly hypomethylated in 2 or more patients showed enrichment of network functions in lipid metabolism, small molecule biochemistry, cell cycle, genetic disorders, cancer, DNA replication, recombination, and repair (Figure 5A and 5B). For example, ARHGDIA is a Rho GDP-dissociation inhibitor, Sox5, 12 and 13 regulate transcription. ULBP1 and ULBP2 activate natural killer cell and regulate immune response. MAPK, Stat 5a/b, Notch, and NF-kB JAK, RAS, PI3K, P38 MAPK, RAS homologue, ARHGEF families, and WNT3 are associated with cell death, apoptosis, cell survival, proliferation, and migration. RNAase has binding activity and regulates transcription; CDKN1A regulates cell cycle. It is likely that secondary resistance to DAC is associated with those downstream pathways.

### Cytogenetic progression

We analyzed cytogenetic data of MDS patients with primary and secondary resistance. For those 32 patients involved in the study of primary resistance, 9 responders had normal cytogenetics, 4 showed abnormalities including translocation of 11 to 20, deletion of chromosome 5, 10, 11,16 and 20, trisomy19, 20, and 3 had no data. By contrast, 4 non-responders had normal cytogenetics, 9 showed abnormalities including 5q 13q, 33, deletion 7 and 19, trisomy 8, 9, and 21 as well as complex changes, and 3 had no data (Table 2). Two non-responders who had poor cytogenetics actually had a low CDA/DCK ratio at 0.25 and 0.53, respectively, suggesting that high CDA/DCK is a risk factor for resistance to DAC, but low CDA/DCK may not be able to overcome bad karyotypes. For those patients involved in the study of secondary resistance, at diagnosis, 15 patients had normal cytogenetics. Of these, three patients showed cytogenetic progression at relapse (new abnormalities included trisomy 8, deletion 16q, and complex chromosome changes). 15 patients had abnormal cytogenetics at diagnosis. Of these, 12 had the same abnormalities at relapse, and 3 had additional changes at relapse (deletion of chromosome 7 in 1 patient and complex changes in 2 patients). Thus, 6 out of 30 (20%) of patients had evidence of cytogenetic progression in secondary resistance (Table 2).

**Table 2:** Cytogenetic progression in MDS patients.

Primary Resistance (Unpaired)		Secondary Resistance (Paired)		Time (months) between diagnosis and relapse
Responders	Non-responders	Diagnosis	Relapse	
+19, +20	5q, 13q, 33, −7, +21	Normal	+8	7
−5, −10, −11, −16, +19, +20	+8	Normal	Del 16 q	10
−20, translocation 11 to 20	5q	−5	−5,−7	21
Complex	+18, +19	−5, −7	+5, −5, −7, +8, −15, −18, −21, −22	12
	−7, −19	Normal	Complex	24
	−7	5q-, −7, −11, and −20	Complex	14
	Complex			
	Complex			
	Complex			

9 non-responders with primary resistance showing abnormal cytogenetics, 6 patients showing cytogenetic progression from diagnosis to relapse.

## Discussion

The two hypomethylating agents DAC and azacitidine have received FDA approval for the treatment of MDS. However, it remains unclear why some patients are resistant to treatment. Our results show that primary resistance to DAC could be related to a higher ratio of CDA/DCK in a subset of patients, which means DAC is less activated through mono-phosphorylation by DCK and more inactivated through deamination by CDA in non-responders. Secondary resistance is likely due alternate progression pathways as we found less aberrant DNA methylation than at diagnosis, and there were no significant changes in DAC metabolism gene expression.

Mechanisms of in-vivo resistance to nucleoside analogues are complex and remain unresolved. One possibility might result from insufficient intracellular triphosphate, which has been tested for a number of drugs such as cytarabine, fludarabine, and 2-CdA in different trials [6]. However, it remains experimentally very difficult to test this for DAC because clinical treatment is at low doses and its incorporation is at very low levels. Unlike the traditional cytotoxic therapies that induce rapid responses in MDS (mostly after one cycle), DAC has a different pattern of responses, which are rare after one cycle and improve over time [20], [21]. In humans, DAC has a short half-life (minutes) due to rapid inactivation in the liver by cytidine deaminase [22], [23]. Therefore, an alternate way to study DAC incorporation/activation is to measure gene expression related to its metabolic pathways as in our previous study in-vitro in cancer cell lines [8]. Here, we found that the CDA/DCK ratio was statistically higher in non-responders than responders. These data favor a pharmacological mechanism of primary resistance for a subset of patients. The data on DCK are particularly relevant clinically given that azacitidine uses a different enzyme for initial mono-phosphorylation; thus, some patients with primary resistance to DAC could benefit from a therapeutic trial with azacitidine. However, multiple mechanisms must be active in different patients as we also found low CDA/DCK levels in some patients with primary resistance, that might not be able to overcome downstream pathways to resistance to DAC such as aberrant chromosome changes or defective induction of apoptosis, and others.

Secondary resistance to hypomethylating agents is emerging as a serious clinical problem. Survival at relapse after an initial response is poor. Here, we investigated secondary resistance using paired diagnosis/relapse samples and find that it is unlikely to be due to pharmacological mechanisms. We previously found that in-vitro acquired resistance to DAC in an HL60 cell line was due to DCK gene mutations [8], which also give rise to resistance to other NAs in other cell lines [24], [25], [26], [27], [28], [29]. However, DCK mutations were not detected in patients after relapse. Similarly, DCK mutations were rare in clinical resistance to other NAs [30], [31]. Although we found that the CDA/DCK ratio was higher in primary resistance to DAC, there was no significant difference in expression of these or other relevant genes between diagnosis and relapse in this study. The role of gene expression related to metabolic pathways in secondary resistance to NAs remains controversial. Some have observed a significant correlation between these gene expression or protein expression and clinical outcome to NA with relapsed and/or refractory leukemia. Conversely, other authors did not find this kind of relationship [6].

Another line of evidence against a pharmacologic mechanism for secondary resistance is the absence of hypermethylation at relapse. In fact, we observed that patients had significant hypomethylation at relapse compared to diagnosis, which cannot be explained by differential blast counts or other obvious confounders. Previously, we found that hypermethylation is accentuated in AML after relapse [12] when patients received traditional chemotherapy containing cytarabine combinations. Thus, it is likely that hypomethylation induction by DAC does not recover in the face of continuing treatment, and that hypomethylation does not prevent patients' relapse and progression. Indeed, one cannot exclude the possibility that hypomethylation itself might eventually lead to progression and resistance to DAC either through ectopic gene reactivation or by mutagenesis and induction of chromosomal instability. Moreover, clinical responses to hypomethylating drugs in-vivo are complex and may involve differentiation and immune activation components. The bone marrow microenvironment is also an important factor to modulate response to chemotherapy [32]. Thus, secondary resistance to DAC may also arise by complex mechanisms not entirely related to initial drug disposition.

Cytogenetic analysis showed that MDS patients after relapse showed evolution in 20% patients with abnormalities such +8, deletion of 16q, and −7. Cytogenetic evolution in MDS has been associated with progression to AML, and the new abnormalities we observed are already recognized as accompanying patients with poor prognosis, especially those involving loss or rearrangements of chromosome 7 and gain of chromosome 8. There are two broad critical regions of deletion on the long arm of chromosome 7 at bands 7q22 and 7q34-q36, which may contain important tumor suppressor genes that could be related to prognosis and resistance to DAC. This issue should be studied further using high resolution chromosomal analysis (for example by SNP-arrays) and/or genome sequencing to identify novel mutations in this setting. Overall, our data suggest that evolution to a more aggressive clone that is perhaps less dependent on DNA hypermethylation for survival may be a common mechanism of secondary resistance to decitabine.

In conclusion, we found that a high CDA/DCK ratio may be a marker of primary resistance to DAC in a subset of patients. If confirmed in other studies, this may help predict response to DAC treatment based on the value of CDA/DCK, or may steer patients towards azacitidine therapy, which does not depend on DCK for activity. By contrast, secondary resistance to DAC is likely independent of DNA methylation and pharmacologic pathways. It is more likely that genetic activation of oncogenic survival and progression pathways contribute to secondary resistance to DAC.

## Supporting Information

Table S1 Primers for pyrosequencing-based analysis.
(DOC)

Table S2 Genes hypomethylated in 4/4 and 3/4 patients after relapse.
(DOC)

Table S3 Genes hypomethylated in 2/4 patients after relapse.
(DOC)

Table S4 Genes hypermethylated in patients after relapse.
(DOC)
