# Supplementary material for: Mechanisms of Resistance to Decitabine in the Myelodysplastic Syndrome
Source: PLoS One. 2011 Aug 17;6(8):e23372. doi: 10.1371/journal.pone.0023372 (PMC3157379; doi:10.1371/journal.pone.0023372)
Supplement: Table S1 — Primers for pyrosequencing-based analysis. (DOC) [file pone.0023372.s001.doc]

| **Supplementary Table 1.** Primers for pyrosequencing-based analysis | | | | | |
| --- | --- | --- | --- | --- | --- |
| Gene | Distance to TSS | Step | Primer name | Sequence ' 5 '3 | '5 - modified |
| NOR1  OLIG2  P15  CDH13  PGRA  PGRB  LINE  MAPK  15  miR-124a-1  miR-124a-3 | -37 to -20  -66 to -41  +127 to +147  +74 to +102  +267 to 329  +868 to 893  -89 to +3 | I  II  S  I  II  S  I  II  S  I  II  S  I  II  S  I  II  S  I  S  I  S  I  II  S  I  II  S | Nor131F1  Nor131 R  Nor131RU Nor131F2  Bio-Uni NOR131S  Olig209F Olig209R Olig88R2U Olig209F  Bio-Uni OLIG209Sgat  P15-314F P15-314R P15-159F2 P15-159R2Bio P15-159S  CDH13-186F CDH13-186R CDH13-101F2 CDH101R2-Bio CDH13-S3F2  PgrA252F PgrA205R PgrA205RU PgrA-F2 Bio-Uni PgrA-S8  Pgr207F Pgr207R  PgrBF3  PgrB R-Bio Pgr207-S4F  LINE-NF LINE-NR-Bio LINE-NS  F  R  R-bio  F  R  F2  R2-Bio  F  R  F2  R2-Bio | GAGTTGGATTGGTGAAGAGTT  ACCAAACCCCCTTCTAATT  GGGACACCGCTGATCGTTTACCAAACCCCCTTCTAATT  GGGGTTAGGTAGTTAGTGG  GGGACACCGCTGATCGTTTA  TTAGGTTGTTGGGGTAA  TTTTAAAGGTGAGGATGTTTATTAT  AAAAATCCAAACCCCCTATAT  GGGACACCGCTGATCGTTTACTCCCTCCCAAAAACCTCAA  TTTTAAAGGTGAGGATGTTTATTAT  GGGACACCGCTGATCGTTTA  GTGAGGATGTTTATTATAGAT  AGTTTAAGGGGGTGGGGAGA  AAAACCTAAACTCAACTTCATTACCC  TTAGAGAGAGGAGGGGTAGTGAGGATT  TCCTTAACTCCCAACTTTTCCTAAC  TTAGGAGTTTTTTTTTAGAAGTA  TTTGGGAAGTTGGTTGGTTG  ACAACCCCTCTTCCCTACCT  AGTTTGGTTTTTAAGGAAAATATGTTTAGT  AACCAAATTCTCCACTACATTTTATCC  AGGAAAATATGTTTAGTGTA  GGAGGAGAAAAGGGGAGTTTA  AAATCCTATCCCTAACAAAA  GGGACACCGCTGATCGTTTAAATCCTATCCCTAACAAAA  ATTGAGTTGAAGGTAAAGGGTTT  GGGACACCGCTGATCGTTTA  GGATTTTTATTGTTGTGT  TGTGGGTGGTATTTTTAATGAGA  CCCCCTCACTAAAACCCTAAA  GAGAATTAGTTTTATTTGTTATTTGAGTGA  CAACCCATTCCCAAAAAAAATC  GGGATTTGAGATTTT  TTTTGAGTTAGGTGTGGGATATA  AAAATCAAAAAATTCCCTTTC Biotin  S AGTTAGGTGTGGGATATAGT  TTTGAGTAGGTATTTTTGGATAAT  CCACAACCCACTCAACAATAA GGGACACCGCTGATCGTTTAGTAGGTATTTTTGGATAATG CCACAACCCACTCAACAATAA  AAAGGTGAAAGAAAGGAAGAGG  TCTCCCACTTCCACCCACA  GGGTGGGTAGAAGATGGAATAA GGGACACCGCTGATCGTTTACCACCAAAAAAATACTATAATCCC AATTAGGAAAAAGAAATAAA  GGGAGAAGTGTGGGTTTTTT  CCTTAATTATATAAACATTAAATCAAAATC  GGGAGGATTGGGATAGTAT  GGGACACCGCTGATCGTTTAAACCTCCAAACCAAAAT GGATTGGGATAGTATAG | Biotin  Biotin  Biotin  Biotin  Biotin  Biotin  Biotin  Biotin  Biotin  Biotin |

Note: F means forward primer; F2 Forward primer 2; R means reverse primer; Bio means universal biotin-labeled primer; Bio-R means Biotin-conjugated primer.
