# Supplementary material for: Mechanisms of Resistance to Decitabine in the Myelodysplastic Syndrome
Source: PLoS One. 2011 Aug 17;6(8):e23372. doi: 10.1371/journal.pone.0023372 (PMC3157379; doi:10.1371/journal.pone.0023372)
Supplement: Table S2 — Genes hypomethylated in 4/4 and 3/4 patients after relapse. (DOC) [file pone.0023372.s002.doc]

| **Supplementary Table 2.** Genes hypomethylated in 4/4 and 3/4 patients after relapse | | | |
| --- | --- | --- | --- |
| 4/4 patients | 3/4 patients | | |
| ESRP1 | | PQLC2 | | --- | | C10orf57 | | SERINC4 | | HSBP1 | | ACBD4 | | ERF | | LOC440925 | | SEC14L2 | | RAET1L | | CCND3 | | NUDCD3 | | ADCK5 | | COBRA1 | | TXNIP | | | ADRA2A | | --- | | KCTD12 | | SMPD3 | | SMCR7 | | SHKBP1 | | FBXL12 | | RASL10A | | DPYSL3 | | MDFI | | SLC4A2 | | PUF60 | | SH2D3C | | SLFN5 | | PRPF4B | | | PDZD7 | | --- | | ELMOD1 | | NUBP2 | | GINS2 | | TSSK6 | | C19orf70 | | DNAJB2 | | TMEM115 | | NQO2 | | DSP | | RAD21 | | PPP3CC | | PPAP2B | | TMTC2  ENOSF1 | |
