# Supplementary material for: Mechanisms of Resistance to Decitabine in the Myelodysplastic Syndrome
Source: PLoS One. 2011 Aug 17;6(8):e23372. doi: 10.1371/journal.pone.0023372 (PMC3157379; doi:10.1371/journal.pone.0023372)
Supplement: Table S3 — Genes hypomethylated in 2/4 patients after relapse. (DOC) [file pone.0023372.s003.doc]

| **Supplementary Table 3.** Genes hypomethylated in 2/4 patients after relapse | | | | | | |
| --- | --- | --- | --- | --- | --- | --- |
| ARHGAP2  FAIM2  WNT3  PFKFB4  C1orf183  CAMK2G  LEPREL2  RHOB  SV2C  NPC1  BCL7C  FGD6  TACR1  FARS2  ZNF557  UBE2J2  MFSD4  EPB41  KLHL21  ATE1  SPON1  MAP3K11  TBX5  MTMR6  STRN3  ZNF770  SNUPN  SLC6A2  OVCA2  MBTD1  NT5C  CACNA1A  DOT1L  ARHGEF1  CDCA7  ADI1  RASSF2  TOM1  POLR2H  KIAA0922  EBF1  CNKSR3  SLC17A5  KIAA0895  MGC70857  IKBKB  HSPA5  ENDOG | FGFR2  COL2A1  ZNF263  LIMD1  C9orf72  SFMBT2  KCNA1  HPCAL1  C4orf42  DUT  EIF3J  MTMR2  ATG9A  C6orf226  CNN1  MTHFR  DSTYK  WASF2  ROR1  TIAL1  TBCEL  C11orf2  IFT81  RAD52  PRKD1  ZC3H14  CPLX3  TGFB1I1  ZNF778  CROP  ARMC7  DNASE2  SFRS14  DPP9  ZNF561  GALNT14  DBNDD2  LOC642852  MED12L  RAB28  SPRY4  ULBP1  KCNQ5  KLHL7  FBXL6  ZNF703  RNH1 ASTN2 | S100A13  PDLIM1  ARHGEF7  CNNM3  TCTE1  PTPRU  GPR137  ITPRIPL2  EMID1  CAV1  C11orf82  VIPR1  HIRIP3  LRBA  CEP350  AHCYL1  RUSC1  MIXL1  MTF1  PAP2D  TYSND1  HSD17B12  ARHGEF17  SPRYD4  WDR20  WDR89  FBN1  ADAMTS17  RSPRY1  CCT6B  TACO1  MOCOS  GPSN2  KIRREL2  ZNF321  TUBA4A  CNNM4  BMP2  ADM2  SLC25A20  KIT  RGMB  C6orf27  MET  GNAI1  POLR3D  SDC2  NTNG2 | C1orf93  TP53I11  MESDC1  ITPA  CAV1  SFXN4  TM7SF2  BCKDK  KLHL24  SLCO5A1  SOX5  RCHY1  INO80E  UTP3  PARD6G  KCNA3  ALDH4A1  MRPL55  SF3A3  COX15  RNLS  CD82  RSF1  NAB2  ANG  PSEN1  SNX22  BFAR  DOK4  DDX52  C17orf100  PSTPIP2  TPM4  DPF1  POLRMT  EFHD1  SOX12  CBS  PHLDB2  MANF  LIN54  SNX3  CDKN1A  ARL4A  PEX1  SLC39A14  ABCA1  XKR5 | PTPRU  GPR137  ITPRIPL2  EMID1  AUTS2  FGFR2  COL2A1  ZNF263  LIMD1  C9orf72  STK38L  PPM1K  RPGRIP1L  TBC1D7  C1orf163  SDF4  RABIF  ARID4B  PODN  ATRNL1  YAP1  PACSIN3  ST5  C12orf61  ABHD4  PAPLN  MEGF11  HAGH  PLA2G15  STAT5A  FADS2  ZCCHC2  USE1  GGN  RPS9  PASK  TOP1  PDXK  RPN1  C3orf38  ROPN1L  REV3L  MED20  CALU  NOV  TNFRSF10B  FSD1L  NTNG2 | SFXN4  TM7SF2  BCKDK  KLHL24  SLCO5A1  ARHGAP21  FAIM2  WNT3  PFKFB4  C1orf220  ISCA2  C9orf93  NGFR  FNDC1  CRKRS  IGSF3  SOX13  LIN28  OMA1  SLC18A2  USP47  RCOR2  FOXN4  KITLG  PCK2  TMED8  PIAS1  RNPS1  C16orf46  SNF8  SLC39A11  ICAM1  OCEL1  C19orf20  KISS1R  FOXN2  JPH2  C21orf29  NMNAT3  MAEA  NEUROG1  ULBP2  FAM83B  PARP12  GPAA1  TNFRSF10D  CDC26 | SFMBT2  KCNA1  HPCAL1  C4orf42  S100A13  PDLIM1  ARHGEF7  CNNM3  TCTE1  ZNF224  C17orf45  ZFP36L1  ABCC5  TRUB2  IER3  PRPF3  DDOST  CCDC28B  GIPC2  PFKFB3  DKFZp686O24166  PPFIA1  LRP6  INTS6  SNX6  IFI27L2  HOMER2  NUDT21  CDK10  TRIM25  SLC38A10  ZSWIM4  GRAMD1A  SLC1A5  TMEFF2  CCDC88A  PPP1R3D  CDC42EP1  GORASP1  RBPJ  RNF130  RPP21  LRCH4  PSMA2  SH2D4A  SLC1A1  MRPS2 |
