# Supplementary material for: Mechanisms of Resistance to Decitabine in the Myelodysplastic Syndrome
Source: PLoS One. 2011 Aug 17;6(8):e23372. doi: 10.1371/journal.pone.0023372 (PMC3157379; doi:10.1371/journal.pone.0023372)
Supplement: Table S4 — Genes hypermethylated in patients after relapse. (DOC) [file pone.0023372.s004.doc]

| **Supplementary Table 4.** Genes hypermethylated in patients after relapse | | |
| --- | --- | --- |
| 4/4 patients | 3/4 patients | 2/4 patients |
|  | NQO2  PTGES2 | LOXL1  PTGES2  SAT1  PANK4  MLLT10  DUT  CCDC154  IL34  QRICH2  SLC17A7  RTKN  SCARF2  MOV10L1  CHN2  NKX6-3  WDR13  RUSC1  HOXA5  TGIF1  CD70  WDR32 |
